# Single-molecule imaging of electroporated dye-labelled CheY in live *Escherichia coli*

**DOI:** 10.1098/rstb.2015.0492

**Published:** 2016-11-05

**Authors:** Diana Di Paolo, Oshri Afanzar, Judith P. Armitage, Richard M. Berry

**Affiliations:** 1 Biological Physics Research Group, Clarendon Laboratory, Department of Physics, University of Oxford, Oxford OX1 3PU, UK; 2 Department of Biological Chemistry, The Weizmann Institute of Science, 7610001 Rehovot, Israel; 3 Department of Biochemistry, University of Oxford, Oxford OX1 3QU, UK

**Keywords:** electroporation, single-molecule fluorescence microscopy, chemotaxis, bacterial motility

## Abstract

For the past two decades, the use of genetically fused fluorescent proteins (FPs) has greatly contributed to the study of chemotactic signalling in *Escherichia coli* including the activation of the response regulator protein CheY and its interaction with the flagellar motor. However, this approach suffers from a number of limitations, both biological and biophysical: for example, not all fusions are fully functional when fused to a bulky FP, which can have a similar molecular weight to its fused counterpart; they may interfere with the native interactions of the protein and the chromophores of FPs have low brightness and photostability and fast photobleaching rates. A recently developed technique for the electroporation of fluorescently labelled proteins in live bacteria has enabled us to bypass these limitations and study the *in vivo* behaviour of CheY at the single-molecule level. Here we show that purified CheY proteins labelled with organic dyes can be internalized into *E. coli* cells in controllable concentrations and imaged with video fluorescence microscopy. The use of this approach is illustrated by showing single CheY molecules diffusing within cells and interacting with the sensory clusters and the flagellar motors in real time.

This article is part of the themed issue ‘The new bacteriology’.

## 1. Introduction

Many species of motile bacteria use rotating extracellular filaments to propel themselves through liquid media. Each filament is driven by a membrane spanning rotary nano-machine called the bacterial flagellar motor. In *Escherichia coli*, the motor is powered by a transmembrane flux of H^+^ and the electrochemical energy is converted into work through a ring of stator units pushing on a central rotor. *Chemotaxis* is the biasing of movement towards regions that contain higher concentrations of beneficial, or lower concentrations of toxic, chemicals, and is one of the best understood bacterial sensory pathways. In *E. coli*, chemotactic signalling is mediated by the phosphorylation of the response regulator protein CheY. CheY ∼ P transduces changes of environmental chemical gradients, detected by specific transmembrane chemoreceptors, to the flagellar motors by binding to the C-ring of the motor and inducing a cascade of conformational changes that modulate the direction of rotation.

To perform an accurate study of the interactions between chemotaxis proteins and the bacterial flagellar motor, it is essential to investigate their behaviour *in vivo*. Most current research relies on the use of fluorescent proteins (FPs). Over the past 20 years, these have revolutionized cell biology via their use as genetically encoded protein labels. Green fluorescent protein (GFP) [1–3] and its mutated allelic forms, blue, cyan and yellow FPs are expressed as fluorescent chimeric proteins in living cells, tissues and entire organisms, after transfection with the engineered vectors [4–6]. Red FPs have also been isolated from other species, including coral reef organisms, and are similarly useful [7,8]. The fluorescent protein technique avoids the problem of purifying, labelling and introducing proteins into cells or the task of producing specific antibodies to surface or internal antigens. These fluorescent fusions have allowed studies of the copy number, diffusion patterns and intracellular localization of proteins involved in processes such as gene expression or membrane transport [9–11]. In the bacterial flagellar motor, they have been successfully used to measure stoichiometry of different components in single motors, leading to the discovery that both rotor and stator proteins exchange on a timescale of minutes with cellular pools [9,12,13]. However, this method has limitations. (i) Being genetically encoded, all target proteins are labelled, which can be an advantage for some applications such as, for instance, estimation of cytoplasmic concentration, but is less good for tracking single molecules; (ii) FPs are much less bright (sixfold for GFP) [14,15] and photo-stable (100-fold for GFP) [16] than commercially available small organic dyes and are, therefore, far from ideal for single-molecule tracking purposes; (iii) because the FPs are relatively large, not all fusions are fully functional, limiting the interpretation of the data and the proteins available for study.

For many years researchers have been trying to find smaller, more stable fluorophores that could be used inside living cells. Organic dyes remain the prime choice for *in vitro* experiments due to their greater photostability, small size (up to 100-fold smaller volume than FPs) and ease of intramolecular labelling (mainly through the use of cysteine residues) [14,15]. All these factors are particularly important for single-molecule fluorescence imaging and tracking. However, as these are not compatible with genetic fusion, a means for re-introducing externally dye-labelled proteins into cells is required for *in vivo* studies. Several internalization methods combining the advantages of organic labelling and *in vivo* detection have been introduced over the past decade, some employing relatively large polypeptide tags [17,18] that are not ideal for bacterial application, while others were limited to large, single-membrane eukaryotic cells (e.g. scrape loading, syringe loading, microinjection) [19–22]. The internalization method in this work, adapted from Crawford *et al*. [16], is based on electroporation where the application of an external electrical field to a low ionic strength cell suspension creates transient pores in the membrane, allowing the uptake of DNA or other macromolecules. Combined with cysteine-maleimide dye labelling and single-molecule fluorescence microscopy, this is an extremely powerful tool to investigate protein intracellular behaviour and interactions for a wide variety of moieties in an even wider range of bacterial species.

In this work, we showcase the applicability of such a technique to the bacterial flagellar motor and chemotaxis fields by imaging and tracking single CheY molecules travelling between the chemoreceptor clusters and the motors in real time.

## 2. Material and methods

### (a) Plasmids and strains

A *cheY* gene containing 5′ insertion mutation for cysteine before the wild-type sequence, (*cys*)*cheY*, was cloned into the enterobacterial expression vector pFloat [23,24], derived from pET30a (Novagen) and engineered to include in frame of its 5′ region a 6xHis (His6) sequence, a small ubiquitin-like modifier (SUMO) solubility tag and a human rhinovirus 3C protease cleavage sequence (LEVLFQ/GP). Formation of correct construct was checked by sequencing. Both His6- and SUMO tags were removed after protein purification using PreScission Protease, a genetically engineered fusion protein consisting of human rhinovirus 3C protease and GST (glutathione *S*-transferase) which specifically cleaves between the Gln and Gly residues of the recognition sequence LeuGluValLeuPheGln/GlyPro. The plasmid encoding (*cys*)*cheY* was transformed into the *E. coli* strain BL21(DE3) (Novagen) and cells were grown with shaking at 225 r.p.m. at 37°C in Luria-Bertani broth (LB) medium supplemented with kanamycin up to OD_600_ = 0.6 before inducing expression.

A *cheY* gene containing a 3′ insertion mutation for cysteine, *cheY*(*cys*), was cloned in a pQE60-His6Tag vector. A Cys was inserted after the last amino acid in the wild-type CheY sequence (M129), but before the in-frame 6xHis sequence of pQE60. Formation of the correct construct was checked by sequencing. A recombinant pQE60-derivative plasmid encoding for *cheY*(*cys*) was transformed into *E. coli* M15[pREP4] competent cells by heat-shock, and cells were grown with shaking at 225 r.p.m. at 37°C in LB medium supplemented with ampicillin up to OD_600_ = 0.6 before inducing expression.

The JPA1814 *E. coli* strain used in the single-molecule experiments was a derivative of RP437 containing a FliM-YPet fusion (constructed as described in [12]) and sticky filaments phenotype (various regions in the *fliC* gene truncated so that hydrophobic regions are exposed), wild-type for motility and chemotaxis. Electrocompetent cells were grown aerobically with shaking at 225 r.p.m. in tryptone broth (TB) medium at 30°C according to a slightly modified version of the traditionally adopted protocol from Sambrook & Russell [25] with the intention of preserving cell motility. The latter in the final electrocompetent aliquots was checked by microscopic examination and confirmed.

The UU2689 strain, derived from RP437 and featuring a cyan fluorescent protein (CFP) genetically fused to CheZ, was used for the co-localization experiments described in §3d.

### (b) Preparation of protein samples

#### (i) CheY expression and purification

(Cys)CheY expression was induced at OD_600_ = 0.6 by isopropyl-β-d-1-thiogalactopyranoside (IPTG; 0.025 mM final concentration) at 18°C with shaking at 180 r.p.m.; CheY(Cys) expression was induced at OD_600_ = 0.6 by IPTG (0.1 mM final concentration) at 30°C with shaking at 225 r.p.m. For both proteins, after approximately 16 h incubation cells were harvested and resuspended in lysis buffer (50 mM Tris–HCl, 150 mM NaCl, 25 mM imidazole, 10% glycerol, pH 8) in the presence of 1 mM dithiothreitol (DTT; a disulfide bond reducing agent), one tablet of SIGMAFAST™ Protease Inhibitor Cocktail EDTA-Free, and benzonase nuclease (Sigma-Aldrich). Resuspended cells were disrupted by sonication while chilled on ice. The resulting cell lysate was cleared by both centrifugation at 35 000*g* for 45 min at 4°C and by 0.45 µM filtration. A nickel affinity chromatography column was prepared by pouring 1.5 ml Ni-NTA agarose slurry (Bio-Rad) into a chromatography drip column, allowing settlement for 10 min and equilibrating with 30 ml of lysis buffer. The filtered cleared lysate was applied to the column and run through. Protein was allowed to bind and the column was washed with at least 50 ml of lysis buffer before being eluted with 10 ml of elution buffer (50 mM Tris–HCl, 150 mM NaCl, 500 mM imidazole, 1 mM tris(2-carboxyethyl)phosphine (TCEP), pH 8).

The TCEP, which is a stronger reducing agent even than DTT, was used to avoid aggregation and oxidation of the cysteine -SH groups. Six 1 ml fractions of the eluate were collected and stored at 4°C. Further purification of proteins was performed by size exclusion chromatography in a HiLoad Superdex S75 16/60 column (GE Healthcare) equilibrated with labelling buffer, a buffer suitable for maleimide dye labelling, with optimal pH for the cysteine–maleimide reaction to occur (50 mM Tris–HCl, 150 mM NaCl, 1 mM TCEP, pH 6.8 at room temperature/7.4 at 4°C). Following purification, the protein was assayed for its purity by SDS-PAGE and concentrated down to 50–100 µM. Aliquots of pure protein were flash frozen and stored at −80°C for future use.

#### (ii) Choice of dye

**Figure 1.**
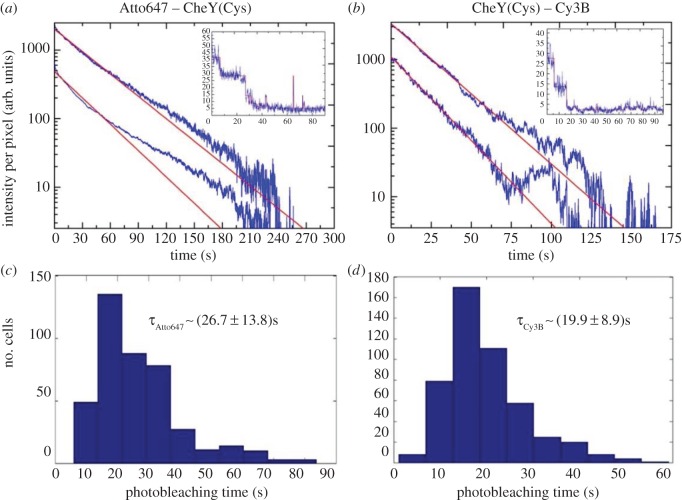
Cell-based photobleaching study of internalized Atto647-(Cys)CheY and CheY(Cys)-Cy3B into live *E. coli* as a measure for photostability. (*a*,*b*) Two example single-cell photobleaching traces over time on a logarithmic scale (blue, raw data) and respective single-exponential fits (red, solid lines). Insets: Single-step photobleaching timetraces from cells loaded with less than six fluorescent molecules (blue) fitted by hidden Markov modelling (red). (*c*,*d*) Histograms of photobleaching lifetimes obtained by the single-exponential fit of single-cell photobleaching traces. Each histogram is based on a dataset consisting of 400–500 cells. Data for both dyes were recorded using nTIRF illumination and 100 ms exposure time; intensity used for Cy3B: 400 nW µm^−2^; intensity used for Atto647: 0.1 µW µm^−2^.

The photostability and brightness of the four organic dyes in table 1 [26–29] were evaluated for their use *in vivo* in *E. coli*. Based on the approach described in [30], the single-cell photobleaching lifetime of cells electroporated with a 1.5 µM CheY(Cys)-Cy3B, Atto647-(Cys)CheY, Atto647N-(Cys)CheY and Cy5-(Cys)CheY was taken as a measure for photostability. The fluorescence decay of the total cell intensity normalized by the cell area was measured and single-cell photobleaching timetraces were obtained. Figure 1*a*,*b* shows such curves on a logarithmic scale for two chosen cells with high and low initial loading (blue). After baseline subtraction, the raw single-cell photobleaching timetraces were fitted with a single exponential (figure 1*a*,*b*, red) and distributions of the estimated lifetimes plotted for all samples. Figure 1*c*,*d* shows the histograms obtained for Atto647-(Cys)CheY and CheY(Cys)-Cy3B. Cy3B had a photobleaching lifetime of (19.9 ± 8.9) s (500 cells), Atto647 of (26.7 ± 13.8) s (400 cells), Atto647N of (29.5 ± 16.8) s (200 cells) and Cy5 of (3.8 ± 1.8) s (200 cells). These are in the range of previously reported values for such fluorophores in similar acquisition conditions [30]. The pool of candidates for protein labelling was, therefore, narrowed down to Cy3B, Atto647N or Atto647 which showed comparable photostability *in vivo*.

**Table 1.** Visual comparison of polarity and hydrodynamic properties of dyes whose maleimide modifications were considered in this work for protein labelling. The evaluation of the fluorophore labelling performance is based on the degree of labelling values obtained for the CheY-dye conjugates and discussed in §2b: ++, very high;+, high; −, low.

dye	labelling efficiency	polarity	hydrodynamic behaviour	reference
Cy3B	++	neutral	highly hydrophobic	[26]
Atto647	+	cationic	hydrophobic	[27]
Atto647N	−	neutral	highly hydrophilic	[28]
Cy5	++	neutral	hydrophilic	[29]

Both Cy3B and Atto647 have been previously shown to have very high brightness *in vivo* when attached to dsDNA [30]. The brightness of Atto647N and Atto647 was considered similar, as the only difference between the two dyes is in their net charge (table 1). Brightness values in the same range as those reported in [30] were found in this work for Cy3B and Atto647 attached to CheY proteins. Single-molecule photobleaching steps in the fluorescence timetraces of *E. coli* cells electroporated with a 15 nM concentration of CheY(Cys)-Cy3B and Atto647-(Cys)CheY were taken as a measure of fluorophore brightness *in vivo*. Single-step photobleaching timetraces from cells loaded with less than six fluorescent molecules were fitted by hidden Markov modelling (HMM) as previously described [16] and the photobleaching step heights were obtained for 45 and 60 cells, respectively (figure 1*a*,*b*, insets: blue curves, raw data; red curves, HMM fits; distributions of step heights in electronic supplementary material, figure S1). Average unitary fluorophore intensities of 3200 photons per second for Cy3B, and 5400 photons per second for Atto647 were found. The total illuminated area for both lasers corresponded to the full camera chip of 512 **×** 512 pixels, i.e. 2316 µm^2^; therefore, the illumination intensities used were approximately 400 nW µm^−2^ for Cy3B, approximately 100 nW µm^−2^ for Atto647 and Atto647N, and approximately 200 nW µm^−2^ for Cy5. Unitary fluorophore intensity is expected to be proportional to illumination intensity, until saturation is reached.

Cy-Dyes are a group of highly fluorescent molecules that cover a wide spectral range and they are used as probes in many biological applications; however, most Cy-Dyes are vulnerable to *cis*/*trans* isomerization about their polymethine linker which leads to loss of fluorescence upon excitation [31]. Cy3B is a conformationally locked version of Cy3, the green emitting dye in this series: it means it is not prone to photo-isomerization and has superior fluorescence properties. The reason why this dye was not chosen for single-molecule experiments is that bacterial cells exhibit a quite pronounced autofluorescence in the green region of the spectrum due to endogenous flavins [32]. The oxidized form of flavin is excited at 450–490 nm and emits at 500–560 nm, whereas the reduced state exhibits no fluorescence. Owing to the aerobic experimental conditions adopted in this work, the presence of some coenzymes in the oxidized, green-fluorescent form in the cell suspension could not be excluded. Cy3B was deemed suitable for the high-loading experiment described in §3d, where the background fluorescence from various sources could be disregarded due to a much brighter signal from the internalized probes; however, a dye emitting in a different channel was selected for single-molecule experiments, where precise measurement of brightness and accurate tracking of a single labelled protein would suffer more from cellular autofluorescence. The ultimate choice of dye ended up, therefore, being between Atto647N and Atto647, both emitting in the red region of the visible spectrum. Diode laser excitation at wavelengths greater than 635 nm and red-absorbing fluorescent dyes were shown to reduce autofluorescence of biological samples and cell damage when working with live cells [33]. Atto647N is a popular dye in single-molecule experiments as it has outstanding brightness and photostability; however, its positive charge and hydrophobicity (table 1) cause molecules labelled with this dye to stick non-specifically to, for example, microscope coverslides or to cell walls when internalized [34,35]. As a consequence, Atto647N has proved difficult for studying single-molecule dynamics *in vivo* [30]. For all these considerations, the dye selected for the single-molecule work was Atto647 (excitation and emission peaks 647 and 669 nm, respectively, extinction coefficient 120 000 M^−1^ cm^−1^).

#### (iii) CheY labelling

Taking advantage of the engineered cysteine residue inserted at the N- or at the C-terminus of CheY, the purified protein was labelled with a maleimide modification of the organic dye Atto647. Both labels worked equally well for the aims of this project. A 10× molar excess of the latter resuspended in dimethyl sulfoxide (DMSO) was added to the purified protein in a reaction volume of 250 µl (for compounds with low aqueous solubility, like most fluorescent dye maleimides, use of organic co-solvent, such as DMSO is essential). DMSO in the reaction solution was always kept less than 3% vol/vol. Labelling reactions were allowed to proceed in the dark overnight at 4°C. The labelled protein thus obtained was separated from the residual unreacted dye by size exclusion chromatography and was then electroporated into JPA1814 cells in a low salinity buffer (50 mM Tris–HCl, 25 mM NaCl, 0.1 mM TCEP, pH 7.5).

### (c) Protein internalization

#### (i) Electroporation

**Figure 2.**
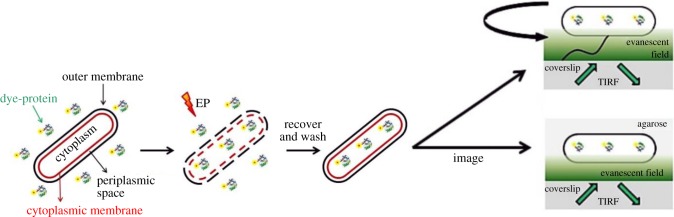
Steps of a typical electroporation experiment: electrocompetent cells are incubated with the fluorescent molecules of interest prior to electroporation; after internalization, they are recovered in rich medium, extensively washed and imaged.

A MicroPulser™ Electroporator (Bio-Rad) was used to apply an electric field of chosen intensity and allow for internalization of dye-labelled proteins of interest. Minimizing any damage caused to cells was the key factor guiding the formulation of the delivery protocol. Figure 2 shows a visual summary of a typical experiment. Electrocompetent cells were incubated with the fluorescent dye-labelled CheY for 5–10 min in a 0.1 cm cuvette; a 12 kV cm^−1^ electric field was applied to allow for internalization of some fluorescent molecules in solution; following electroporation, cells were recovered in super optimal broth with catabolite repression (SOC) at 30°C, 450 r.p.m. in a Thermomixer (Eppendorf) or on 1% EZ rich defined medium agarose pads for an amount of time varying according to the experiment being carried out (minutes to hours). Cells were then washed extensively with motility buffer (MB; 1 M K_2_HPO_4_, 1 M KH_2_PO_4_, 10 mM EDTA, pH 7.0) to remove non-internalized molecules. Alongside the electroporated (EP) sample, a non-electroporated (NEP; positive control) sample, where cells were incubated with the same amount of fluorescently labelled protein as the EP, but to which no electric pulse was applied, and a negative control sample, where cells were never in contact with the fluorescently labelled protein, were prepared. Quantification of CheY fluorescence in samples of intact electroporated cells compared with cellular and supernatant fractions of spheroplast samples, with cell wall and outer membrane removed, indicated that less than 10% of CheY is associated with the periplasm, cell wall or outer membrane (data not shown). Thus, the majority of electroporated CheY is located in the cytoplasm.

#### (ii) Cell visualization

Depending on the experiment, samples for imaging were prepared in two alternative ways: using agarose pads or tunnel slides.

*Agarose pads.* Agarose pads were created by pipetting a droplet (approx. 300 µl) of 1% agarose (BD/Difco) in MB on the centre of a coverslip and leaving it to spread until naturally set. The pad should be raised about 0.5–1 mm from the glass. In the case of internalization and viability tests, MB was replaced with EZ rich defined medium (non-autofluorescent) to ensure cell growth and division. The pad was left to dry for 10–15 min, after which 5 µl of cells was pipetted onto it and a burned^1^ coverslip added on top. The sample was inverted and placed on the microscope with the burned coverslip closest to the objective.

*Tunnel slides.* Tunnel slides for motility studies were made by attaching two strips of double-sided tape (3 M) to the longer sides of a rectangular microscopy slide and placing a glass coverslip on top (electronic supplementary material, figure S2). The glass coverslips used each have a thickness of 170 µm. Solutions were flushed in the tunnel through the small air space between the slide and the coverslip. In the case of *E. coli*, thanks to the FliC ‘sticky’ (FliC^st^) mutation, cells tethered to the glass via their flagella and were incubated in a dark humidity chamber (containing a wet piece of tissue) upside down for 10 min. Non-stuck cells were removed by washing the slide with 100 µl of MB three times, adsorbing liquid from the opposite side with a tissue. The open ends of the tunnel were finally sealed with transparent nail varnish to prevent evaporation and the sample imaged upside down.

#### (iii) Microscopy

Imaging was performed in bright-field and in fluorescence. Cells were visualized on a customized inverted Olympus IX-71 microscope equipped with two lasers, a 637 nm ‘red’ diode laser (Vortran Stradus; Vortran Laser Technology, Sacramento, CA, USA) and a 532 nm ‘green’ Nd:YAG DPSS laser (Samba; Cobolt AB, Solna, Sweden). Laser light was combined into a single-mode optical fibre (Thorlabs, Newton, NJ, USA) and collimated before focusing on the objective. Near-total internal reflection fluorescence (nTIRF) and TIRF illumination were achieved by adjusting the position of the focused excitation light on the back focal plane of the objective (UPLSAPO, 100×, NA 1.4; Olympus, Shinjuku, Tokyo, Japan). Cellular fluorescence was collected through the same objective, filtered to remove excitation light through a long-pass filter (HQ545LP; Chroma, Taoyuan Hsien, Taiwan) and a notch filter (NF02–633S; Semrock, Rochester, NY, USA), and spectrally separated by a dichroic mirror (630DRLP; Omega, Brattleboro, VT, USA). Each channel was imaged onto separate halves of the chip of an EMCCD camera (iXon+, BI-887; Andor, Belfast, UK). The illumination for bright-field images comprised a white-light lamp (IX2-ILL100; Olympus) and condenser (IX2-LWUCD; Olympus) attached to the microscope. Movies and images were recorded using manufacturer's software (Andor). All measurements were carried out in continuous wave (CW) mode for both green and red lasers. Unless differently stated, for all the experiments the exposure time was 10 ms and the excitation power 1 mW for the green laser and 2.5 mW for the red laser. The total illuminated area for both lasers corresponded to the full camera chip of 512 **×** 512 pixels, i.e. 2316 µm^2^; therefore, illumination intensities used ranged between 400 nW µm^−2^ for the green laser and 0.1–1 µW µm^−2^ for the red laser.

Epifluorescence microscopy was mostly carried out on an inverted Nikon Eclipse Ti system connected to an Andor iXON CCD camera. A 100× oil objective (Nikon) was used. The microscope was controlled using NIS Elements software (Nikon). The perfect focus system (PFS) feature was activated when time courses in multiple fields of view were captured in order to maintain a stable focus. For epifluorescence acquisition, a mercury lamp with appropriate excitation and emission filters for the fluorescent dyes being imaged was used.

## 3. Results

### (a) Viability

**Figure 3.**
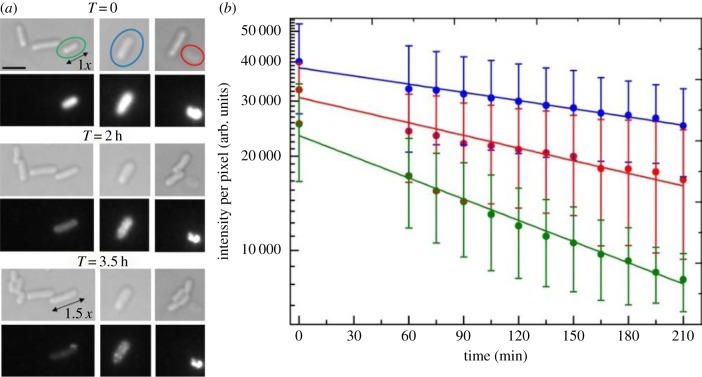
(*a*) Electrocompetent *E. coli* RP437, FliC^st^ cells electroporated with 0.85 µM, i.e. 17 pmols, CheY(Cys)-Cy3B imaged in bright-field (top panels) and in fluorescence (bottom panels) during a 3.5 h time-lapse experiment on rich medium agarose pad. In the first field of view, a cell that has uptaken some fluorescence (green oval) and which increases its size 1.5× by the end of the acquisition is shown. In the second field of view a fluorescent cell that does not grow but remains intact at least until the end of the acquisition, without showing any evident sign of membrane degradation, is shown (blue oval). Finally, in the last field of view, a growing/dividing fluorescent cell (green oval) is shown. (*b*) Average fluorescence decay curves on a logarithmic scale over time for eight growing/dividing (green), four non-growing but intact (blue) and four damaged (red) imaged cells. The single-exponential fit in green yields *τ* = (150 ± 5) min (i.e. approx. 2.5 h) for growing/dividing cells, the fit in blue yields *τ* = (400 ± 20) min (i.e. just over 6.5 h) for non-growing but intact cells, and the fit in red yields *τ* = (260 ± 20) min (i.e. just over 4 h) for damaged cells. Single molecules start being distinguishable in the cells from *T* = 3 h onwards. Settings for Cy3B: epifluorescence, exposure time 800 ms. Scale bar, 3 µm (applies to all bright-field and fluorescence images).

Viable cells can grow and divide on agar plates or in liquid medium. By contrast, dying/dead cells have irreversibly lost their capability of growth and multiplication. Besides these two conditions, some electroporated cells were observed that did not grow or divide but did not appear to have damaged membranes [36]. These cells could be in a viable but stressed state after application of the electric pulse, needing further recovery to resume growth/division and motility. Motor rotation can occur in spheroplasts and in artificially energized cell envelopes [37]. Figure 3 shows one example for each type of condition. Cell samples were imaged in bright-field and in fluorescence once every 30 min, to assess cell viability upon recovery in rich medium after electroporation; 68% of the imaged cells (regardless of loading) resumed growth/division within 1 h of electroporation (figure 3, green), 20% were not growing but intact (figure 3, blue) and 12% were visibly damaged and assumed to be dying/dead (figure 3, red). The right panel in figure 3 shows averaged fluorescence photobleaching curves for each type of cell. The photobleaching lifetimes are similar, indicating that if there is any difference in cytoplasmic chemistry it does not strongly affect the dye photostability. Growing/dividing cells are not significantly less bright than the other two types, with some loaded cells still viable after internalization of hundreds of protein molecules. The damage-minimizing protocols developed for both preparation of electrocompetent cells and electroporation, described in §2c, resulted in an increase with respect to previous values reported in the literature for loaded cells [36] of both the proportion which could grow/divide after electroporation (38% versus 11%) and the proportion which would remain intact but not growing up to 3.5 h from application of the electric pulse (43% versus 32%). A summary of the percentages of viable bacteria upon electroporation is given in electronic supplementary material, table S1.

### (b) Motility assessment and recovery

The use of electroporation for the internalization of proteins into live bacteria is a recent method [16,30,36]. A rotating motor showing interaction with CheY proteins is a direct ‘read-out’ of the integrity of the membrane and of functional chemotaxis control and signal transduction. We therefore set out to develop a procedure where the electroporated cells' motility recovered as quickly as possible. If the exposure to the electric field is sufficiently short and the membrane recovery sufficiently rapid for the cell to remain viable, the effects of electroporation are reversible (known as reversible electrical breakdown; REB). Recovery of the membrane after pulsing is clearly essential to achieving motility. Relatively little is known about the kinetics of membrane recovery after the membrane has been damaged by REB. We found small numbers of cells swimming or rotating within minutes of electroporation, and a recovery time in rich medium (defined as at least 10% of EP and NEP cells swimming or spinning) of approximately 75 min or more. For the EP sample, this is probably due to the electric shock inflicted by electroporation, in terms of either disruption of membrane integrity, of proton motive force, or both. The fact that some of the cells in the NEP sample are non-motile immediately after treatment suggests that, in addition to the effects of electroporation, the washing steps necessary to prepare cells for electroporation might contribute to such delay, perhaps breaking the filaments, disrupting the proton motive force or causing an osmotic shock to the cells owing to the very low salt concentration used.

The observation of rotating cells within minutes of electroporation indicates that the cell membrane and proton-motive force can recover on this timescale, even if there is a longer delay before the majority of cells recover sufficiently to grow and divide. For the experiments in §§3c–e, below, we were interested in motility and chemotaxis rather than growth and division, so we imaged cells after 5 min recovery in rich medium to minimize any possible dilution or degradation of labelled CheY due to cell growth and metabolism. Recovery of motility is significant not only for investigation of the interactions between CheY and FliM in a working motor as proposed in this work, but also for the general application of the electroporation technique to the study of the interactions *in vivo* of a range of other chemotaxis and motility proteins. In fact, previously reported experiments involving such a technique in bacteria [16,30,36] did not consider its effects on cell motility, using a strain (DH5α) with a demonstrated poor-motility phenotype [38].

### (c) Motility and functionality in electroporated *Escherichia coli*

Figure 3 shows that viability depends little upon loading during electroporation. We also assessed the ability of cells to recover a motile phenotype after electroporation. In the single-molecule experiments illustrated in §3d,e, cells had to be imaged on agarose pads for the sake of precise localization of the internalized proteins; it was therefore not possible to infer whether motors were functional. In the following, experiments aimed at explicitly showing CheY's interaction with FliM in rotating tethered motors are reported. For all the experiments described in this section, cells were electroporated, washed in MB, recovered for 5 min in SOC and imaged in tunnel slides following the procedures illustrated in §2c.

#### (i) Motility is independent of loading

**Figure 4.**
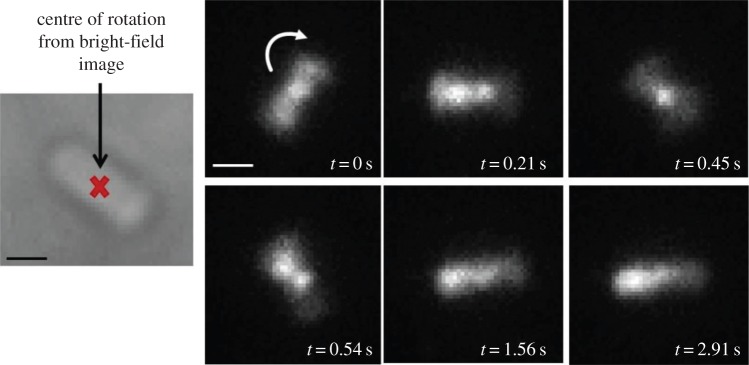
Frames from video fluorescence microscopy performed over 3 s showing single molecules diffusing/binding inside a tethered rotating electrocompetent *E. coli* RP437, FliC^st^ cell electroporated with 1.25 µM, i.e. 25 pmol, CheY(Cys)-Cy3B: at times during the movie (e.g. at *t* = 0 s, *t* = 0.21 s and *t* = 0.45 s), it is possible to clearly see a bright spot on top of the centre of rotation, indicating a possible interaction of CheY with the motor; at other times (e.g. at *t* = 1.56 s and *t* = 2.91 s), the bright spot localizes at one pole of the cell; finally, in some frames (e.g. at *t* = 0.21 s and *t* = 0.54 s), it is possible to observe two bright spots in the cell, one of which is always at the centre of rotation and the other seems to be located at—or travelling to—the cell pole. Exposure time 30 ms, intensity used for Cy3B: 400 nW µm^−2^; intensity used for Atto647: 0.86 µW µm^−2^. Scale bar = 1 µm. (Substack of full video given in the electronic supplementary material, *CheYmot1* movie (20 fps).)

Does uptake of fluorescent molecules upon electroporation affect cell motility on top of the stress caused by the application of the electric pulse itself? Figure 4 shows TIRF images of a tethered electrocompetent *E. coli* cell electroporated with approximately 100 times the amount of CheY protein employed for single-molecule tracking experiments in §3e, i.e. 1.25 µM or 25 pmol. This cell is rotating clockwise at 4 Hz, a speed comparable with the typical speed of tethered wild-type cells (3–10 Hz), proving that motility recovery after heavy loading during electroporation is possible.

#### (ii) Single-molecule interaction with FliM in motile cells

**Figure 5.**
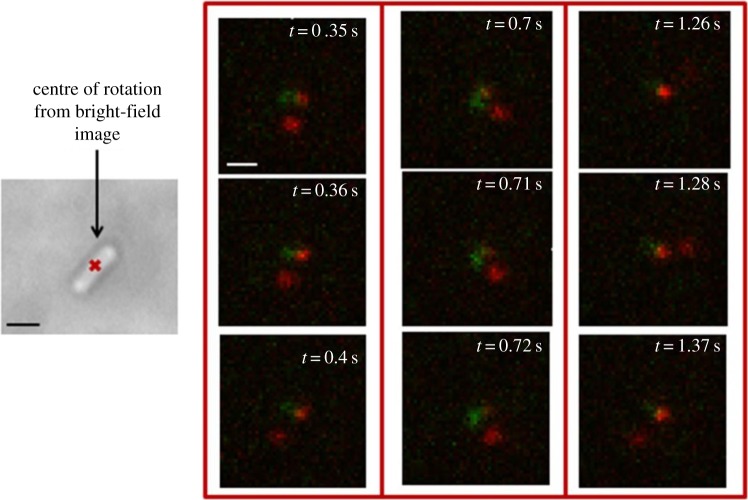
Frames from two-colour fluorescence video microscopy performed over 3 s (substack) showing single-molecules diffusing/binding inside a tethered rotating *E. coli* RP437 (FliM-YPet, FliC^st^) electrocompetent cell electroporated with 20 nM, i.e. 0.4 pmol, Atto647-(Cys)CheY: for most of the movie, it is possible to see very clearly a bright red spot on top of the centre of rotation (green), suggesting a possible interaction of CheY with the motor; at other times (e.g. at *t* = 0.35 s and *t* = 0.71 s), it is possible to see also a second bright spot which localizes at one pole of the cell. Exposure time 10 ms, intensity used for YPet: 400 nW µm^−2^; intensity used for Atto647: 1 µW µm^−2^. Scale bar in bright-field image (in black) = 1 µm; scale bar in fluorescence image (in white) = 500 nm. (Substack of full video given in the electronic supplementary material, *CheYmot2* movie (40 fps).)

Having established that uptake of fluorescent molecules does not affect the electroporated cells more than the application of the electric pulse alone, the interaction of single labelled CheY molecules with motors (and with other possible loci) in real time in a rotating tethered cell was investigated. Figure 5 shows a tethered cell electroporated with Atto647-(Cys)CheY imaged both in bright-field (left) and in fluorescence (TIRF, right): the convention is red for internalized proteins and green for motor spots, labelled through FliM-YPet. Throughout the movie from which the frames in figure 5 are extracted (*CheYmot2*), it is possible to clearly see a persistent red spot overlapping with the green one at the centre of rotation, and another, dimmer red spot at one of the cell's poles.

### (d) Electroporated CheY's functionality

#### (i) Polar localization

**Figure 6.**
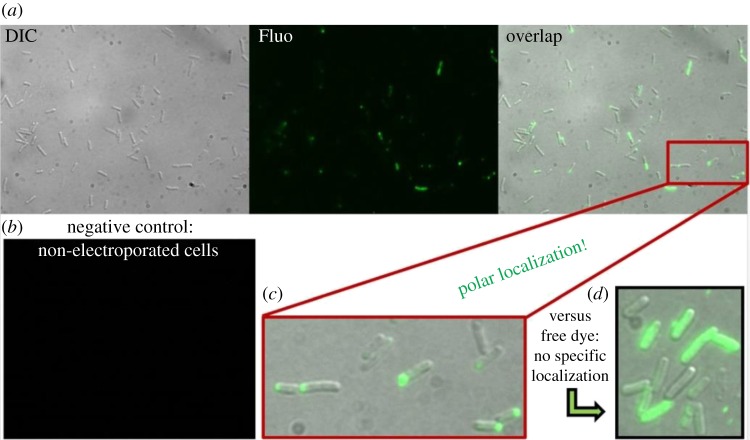
(*a*) Fluorescence images in false colours of electrocompetent *E. coli* cells electroporated with 1.5 µM (30 pmol) CheY(Cys)-Cy3B imaged in DIC, fluorescence and an overlap of the two; (*b*) electrocompetent cells incubated with the same amount of protein but not electroporated showed little or no fluorescence internalization. (*c*) Detail showing polar localization of electroporated CheY-Cy3B proteins inside the cells; (*d*) Further control: electroporated free Cy3B-maleimide dye shows no specific localization. Epifluorescence, 150 ms exposure time. Electroporation parameters and procedure as described in §2c.

Are the re-introduced labelled chemotaxis proteins functional in general, without particular regard to the motor? Figure 6*a* shows a field of view of *E. coli* cells electroporated with 1.5 µM (30 pmol) CheY(Cys)-Cy3B, imaged in differential interference contrast (DIC) and fluorescence. Twenty-six out of 60 observed electroporated cells had internalized some fluorescence, while none was observed in the negative control (cells incubated with the same molecules but not electroporated, figure 6*b*). Fluorescence localized at cell poles, where the chemoreceptors are known to cluster, in 20 of the 26 labelled cells (figure 6*c*). This result, previously reported for wild-type CheY labelled with FPs [39,40], indicates that CheY molecules internalized by electroporation were still intact and retained their function inside the cells. As a further control, cells electroporated with free dye did not show polar localization (figure 6*d*).

#### (ii) CheY–CheZ co-localization

**Figure 7.**
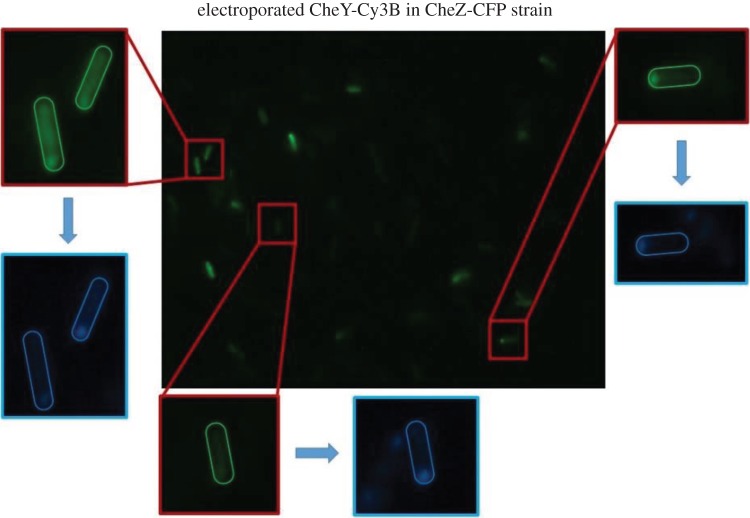
Co-localization of electroporated CheY-Cy3B (green) and CheZ-CFP (blue) in false colours in *E. coli*. Two-colour imaging revealed that Cy3B-labelled CheY molecules localize at the same cell pole as the phosphatase CheZ in electroporated cells showing polar fluorescence. Epifluorescence, 150 ms exposure time, electroporation parameters and procedure as described in §2c.

We investigated co-localization of the electroporated CheY molecules with the phosphatase CheZ, which has been shown to mostly localize at the chemoreceptor cluster at the cell pole [39,41] to verify that the fluorescence at the poles reflects. CheY binding to the chemoreceptors' cluster as previously described, and not an artefact due, for instance, to protein aggregation at the cell poles. Using a strain with a CFP genetically fused to CheZ and two-colour imaging, unambiguous identification of the chemoreceptor cluster was possible. Co-localization of CheY(Cys)-Cy3B and CheZ-CFP in electroporated *E. coli* cells was confirmed in 42% of the cells which showed some degree of fluorescence internalization, as in the example field of view in figure 7. In the remaining 58% of cells, the fluorescence from CheY(Cys)-Cy3B in the imaged cells was very diffuse, and no other locus different from the pole where electroporated proteins tended to localize could be identified. It is assumed that the electroporated CheY is functional, as much bulkier FP fusions show normal rates of *in vitro* phosphorylation. *In vitro* phosphotransfer assays to confirm active phosphotransfer require concentrations of protein much higher than the concentrations of labelled protein we can produce. However, the colocalization result shown in this section suggests that the re-introduced fluorescently labelled CheY was still intact and most probably functional *in vivo* upon electroporation.

### (e) Single-molecule imaging

**Figure 8.**
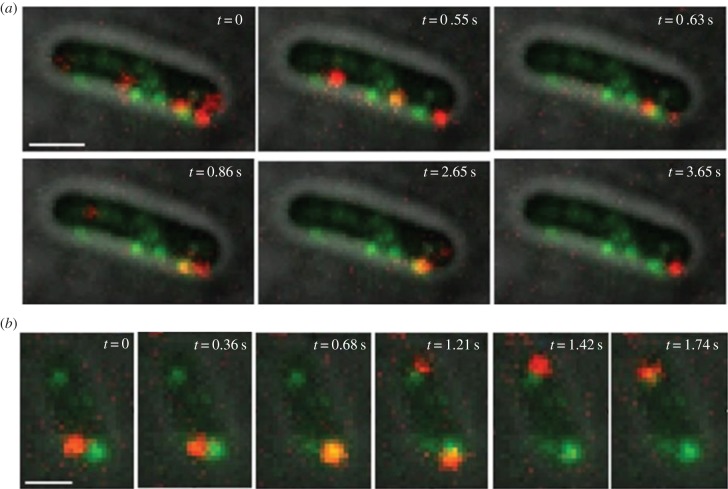
Frames from two-colour video microscopy showing single-molecules diffusing/binding inside *E. coli* RP437 (FliM-YPet, FliC^st^) electrocompetent cells electroporated with 20 nM Atto647-(Cys)CheY. (*a*) In this acquisition, performed over 20 s, CheY mostly localizes with the green motor spots in the cell; it also dwells at some other unlabelled loci which could well correspond to polar or lateral chemoreceptor clusters, as especially visible in the last frame shown (*t* = 3.65 s). Scale bar = 1 µm. (*b*) In this acquisition, performed over 40 s, at *t* = 0 CheY is located next to a motor at the bottom of the cell, travels to it after approximately 550 ms and dwells there for approximately 220 ms; at *t* = 1 s, another CheY molecule co-localizes with the same motor, dwelling there for 230 ms; at *t* = 0.9 s, yet another CheY molecule localizes with the top cell pole, for approximately 800 ms; finally, a last CheY molecule appears at the top motor at *t* = 1.74 s, but disappears after only 20 ms. Scale bar = 500 nm. For both acquisitions: exposure time 10 ms, intensity used for YPet: 400 nW µm^−2^; intensity used for Atto647: 1 µW µm^−2^. (Substacks of full videos given in electronic supplementary material, CheYcoloc1 and CheYcoloc2 movies (20 fps), respectively.)

The working cycle of single CheY molecules *in vivo* was observed and quantified by tracking them in their journey through the cytoplasm between the receptor clusters and the motors. To carry out single-molecule experiments without the loss of resolution caused by overlapping spots, the cellular uptake of labelled proteins upon electroporation was characterized and calibrated to yield internalization of very few molecules per cell (ideally less than five on average). The final labelled protein concentration of 20 nM for electroporation was found to suit this criterion in most experiments. In figure 8, frames from two-colour video microscopy show single molecules of Atto647-(Cys)CheY diffusing/binding inside *E. coli* RP437 (FliM-YPet, FliC^st^) electrocompetent cells. As in figure 5, the convention is motor fluorescence in green and CheY in red.

#### (i) Spot localization and tracking

The fluorescence intensity was corrected for autofluorescence background for each emission channel as described in the electronic supplementary material, figure S3. For each video frame, spots corresponding to internalized labelled CheY molecules were identified as nine connected pixels, all with intensity above a threshold equal to the upper limit of the background fluorescence in the red channel. Each pixel corresponded to 94 × 94 nm. Each CheY spot in each frame was fitted with a two-dimensional Gaussian to determine its location. Single-molecule tracking of CheY was performed by Matlab implementation of a published algorithm [42]. Spots were linked into a track if they appeared in consecutive frames within 10 pixels. Only trajectories more than four frames long were classified as tracks.

#### (ii) Diffusion

**Figure 9.**
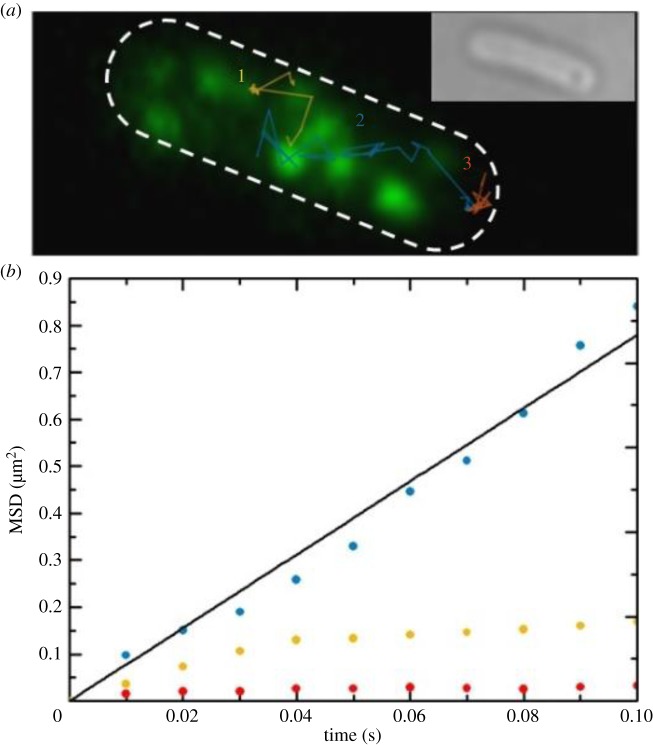
(*a*) Green fluorescent channel image of the same *E. coli* RP437 (FliM-YPet, FliC^st^) electrocompetent cell shown in figure 8*a* electroporated with 20 nM Atto647-(Cys)CheY with its outline in white (dashed line). Three example CheY tracks are superimposed, corresponding to the two main behaviours observed: bound to a locus in the cell which could be a motor (1, orange) and to another unlabelled locus which could be a polar cluster (3, red), or free diffusing in the cytoplasm (2, blue). The inset shows the cell in bright-field. For aesthetic reasons, in this figure the green fluorescence was smoothed by bicubic interpolation, with the output pixel value a weighted average of pixels in the nearest 4 × 4 neighbourhood. Settings: exposure time 10 ms, intensity used for YPet: 400 nW µm^−2^; intensity used for Atto647: 1 µW µm^−2^. (*b*) Mean squared displacement (MSD) curves versus elapsed time over 10 frames (100 ms) for each of the tracks in (*a*) (colours matching tracks). Track 2 (blue) is fit with a straight line through the origin (black), consistent with free diffusion of Atto647-(Cys)CheY at 1.95 ± 0.05 µm^2^ s^−1^.

We electroporated RP437 (FliM-YPet, FliC^st^) *E. coli* cells with 20 nM (0.4 pmol) of Atto647-(Cys)CheY and allowed cells to recover before deposition on agar pads. We imaged YPet using a 532 nm laser, 1 mW of power on the sample, and Atto647 using a 637 nm laser, 2.5 mW of power on the sample in frame transfer mode with an exposure time of 10 ms in TIRF mode. The total illuminated area for both lasers corresponded to the full camera chip of 512 × 512 pixels, i.e. 2316 µm^2^, and the doses used in these experiments were therefore approximately 400 nW µm^−2^ for FliM-YPet and approximately 1 µW µm^−2^ for Atto647(Cys)-CheY. We obtained 1658 single-molecule tracks, 307 longer than 20 frames (200 ms) and 108 longer than 50 frames (500 ms; electronic supplementary material, figure S4), in a total of 48 loaded cells imaged over five sessions with an overall recording time of 33 925 frames. Figure 9*a* shows three example tracks from the movie in figure 8*a*, indicative of the two different behaviours observed for CheY in the cell, confined or freely diffusing: tracks 1 (orange) and 3 (red) are characteristic of the former, probably denoting molecules interacting with a locus in the cell which could correspond to a motor and with the chemoreceptor cluster at the right pole, respectively, whereas track 2 (blue) is characteristic of a molecule freely diffusing in the cytoplasm. These three tracks are all longer than 100 ms, which is the average tumbling time of an *E. coli* cell: namely, track 1 is 400 ms, track 2 is 280 ms and track 3 is 270 ms long. Confirmation of these behaviours comes from the analysis of the mean squared displacement (MSD) curves shown in figure 9*b*, drawn with matching colours to figure 9*a*. The MSD for track 3 does not increase with elapsed time, characteristic of confined movement, whereas that for track 2 is fitted by a line through the origin (black), supporting the hypothesis of free diffusion. Track 1 shows both binding and diffusion, and its MSD represents an average of both types of behaviour. Using the simple relation for MSD in two dimensions, MSD *=* 4*Dt*, with *D* diffusion coefficient and *t* elapsed time, it is possible to estimate a diffusion coefficient for molecule 2 of *D* = 1.95 ± 0.05 µm^2^ s^−1^.

For a statistically significant determination of the nature of a single track we estimate that more than approximately 50 points are required, and an estimate of *D* for CheY in cells requires analysis of many such tracks. We calculated MSD plots similar to figure 9*b* for all 108 tracks longer than 50 frames, and selected the 30 of these for which a line-fit of MSD versus elapsed times from 1 to 4 frames had *R*^2^ > 0.8 as indicative of free Brownian motion. Taking the average slope of these 30 line fits gave 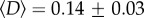 (mean ± s.e.m.) µm^2^ s^−1^ for this subset of CheY molecules.

## 4. Discussion

Imaging of CheY proteins in living cells had previously been performed only via FP-genetic fusions. The low brightness of these probes, however, did not allow for high enough localization precision to perform reliable, prolonged single-molecule tracking and for observation of real-time behaviour. In this work, single CheY molecules labelled with a small organic dye of choice were reintroduced into live *E. coli* by means of electroporation, imaged and tracked through the cytoplasm with 10 ms temporal resolution for up to 1685 frames (16.85 s) in TIRF mode. The half-life of CheY in *E. coli* was estimated with the ProtParam online tool [43–46] as more than 10 h, indicating that it is unlikely that we tracked protein fragments or cleaved dyes in our observations, all of which were made during 30 min to 3.5 h of electroporation. We observed possible interactions with FliM and with other loci in the cell (possibly corresponding to the chemoreceptor clusters) in real time in rotating tethered cells after a few minutes as well as after a few hours' recovery from electroporation in a time-lapse experiment in rich medium. We gave a good indication of their general functionality besides interaction with the motor by performing two-colour fluorescence co-localization studies with CheZ-CFP at the cell poles.

Electroporation allows up to a few hundred molecules to be delivered into each cell. This compares with estimates of approximately 8000 native CheY molecules per cell in the strain we used, under similar growth conditions [47]. Thus, we would not expect electroporated CheY to complement a CheY deletion, and in our experiments, electroporated CheY is not expected to interfere with the function of unlabelled native CheY. Rather, small numbers of electroporated CheY molecules act as representative markers of CheY function in the cell. For proteins with much lower copy number, electroporation may be able to replace the native protein, with the advantage of allowing direct assessment of the functionality of the electroportated labelled protein, but the disadvantage that its function depends on the number of electroporated molecules.

Our estimate of an average diffusion coefficient of 0.14 µm^2^ s^−1^ for CheY molecules that gave long tracks is an order of magnitude or more less than previously reported values. In the literature, a diffusion coefficient of 10 µm^2^ s^−1^ has been previously reported for wild-type CheY *in vitro* [48,49]. To determine CheY's diffusion coefficient *in vivo*, two main techniques have been used: fluorescence correlation spectroscopy (FCS) and fluorescence recovery after photobleaching (FRAP). The former yielded an average diffusion constant of cytoplasmic CheY-GFP fusion of (4.6 ± 0.8) µm^2^ s^−1^ [50]; the latter revealed that CheY had a very fast recovery after photobleaching, on the timescale of several seconds, which could not be clearly resolved from the recovery of the cytoplasmic fraction [51]; the half-time for diffusional recovery (*t*_1/2_) was therefore estimated and used to calculate its effective diffusion coefficient, using the relation *D* = 0.07 *L*^2^/*t*_1/2_, where *L* is the cell length. Values found for CheY were *t*_1/2_ = (0.61 ± 0.12) s and hence *D* = (1.26 ± 0.22) µm^2^ s^−1^ for *L* = 3.3 µm (i.e. the average length of an *E. coli* cell). The discrepancy between these two values can be attributed to the difference in the measurement techniques, with FRAP analysis underestimating the rapidly diffusing fraction of proteins owing to the limited time resolution (0.33 s), and with FCS missing a slowly diffusing fraction. However, in both these methods CheY was fused with a fluorescent protein, which might have affected its functionality and mobility.

Several factors may explain our low estimate of *D*. The first is selection bias for slow molecules due to our relatively long exposure time (10 ms). During one 10 ms frame a particle with *D* = 0.1, 1 or 10 µm^2^ s^−1^ diffuses a root-mean-square distance of approximately 60, 200 or 600 nm, respectively. Given a diffraction-limited spot diameter of approximately 250 nm, molecules with *D* > ∼1 µm^2^ s^−1^ are likely to be missed by our tracking algorithm due to motion blur. In addition, we expect long tracks to be biased towards molecules that associate with or bind to the cell envelope, as those freely diffusing in the cytoplasm will spend relatively short episodes within the TIRF illumination field that extends only a few 100 nm from the surface. Electronic supplementary material, figure S5 shows the distribution of track lengths we recorded, illustrating a preponderance of short tracks that is consistent with transient passage of molecules through the TIRF illumination. Electronic supplementary material, figure S6 shows that *D* falls with increasing track length, possibly indicating that molecules with long tracks are a slow sub-population that is membrane associated. These may be binding to motors or other membrane targets on a timescale that we cannot resolve, or could possibly be among the small fraction of electroporated CheY that may be associated with the periplasm or outer membrane. Other possible reasons for slow diffusion near the membrane include nucleoid exclusion or other biological barriers.

With the electroporation approach described in this work, diffusion properties and kinetics of dye-labelled CheY proteins (and other proteins in general) can be measured for the first time *in vivo* without FP tags. We note that these measurements can be affected by inaccuracies due to confinement within the cell, motion blurring and localization error [51], but a great advantage of the single-molecule approach is that these factors can be modelled and accounted for. Moreover, one of the most important advantages over the use of FPs is that the bright fluorescence signal from the organic dyes and their robustness to photobleaching allows for accurate single-molecule tracking in real time over scales often much longer than the biological processes being investigated, or alternatively in future will allow tracking for relatively long times with the exposure times of 1 ms or shorter that are necessary fully to characterize CheY diffusion.

## 5. Conclusion

The electroporation approach used in this work offers the exciting possibility of a new generation of single-molecule *in vivo* fluorescence experiments in live bacteria. We have demonstrated its potential in the bacterial flagellar motor and chemotaxis fields, but this very powerful technique could be easily applied to a wide range of targets, probes and organisms. A major advantage of the single-molecule approach over batch measurements to investigate chemotaxis is the ability to study the interaction of CheY with the flagellar motor in real time and measure relevant binding constants. Such measurements can be then employed to elucidate the poorly characterized switching mechanism of the flagellar motor. The outcome of these investigations will guide further studies into the detailed effects of chemical stimuli on the dynamics of CheY, the behaviour of other chemotaxis proteins at the single-molecule level and towards understanding of the switching mechanism of the flagellar motor.

## Supplementary Material

Electronic Supplementary Material

## Endnote

1 The procedure for burning coverslips is: ramp up the heat in the furnace at 90°C per hour until 500°C; burn at 500°C for 1 h; cool down at 150°C per hour until room temperature is reached.

## Data accessibility

All data and Matlab scripts used for spot detection and tracking analysis are available upon request. The scripts were composed in the Matlab 2014a environment with functions from the image processing and optimization toolboxes.
