# Supplementary material for: Single-molecule imaging of electroporated dye-labelled CheY in live Escherichia coli
Source: Philos Trans R Soc Lond B Biol Sci. 2016 Nov 5;371(1707):20150492. doi: 10.1098/rstb.2015.0492 (PMC5052738; doi:10.1098/rstb.2015.0492)
Supplement: Electronic Supplementary Material [file rstb20150492supp1.pdf]

Electronic Supplementary Material

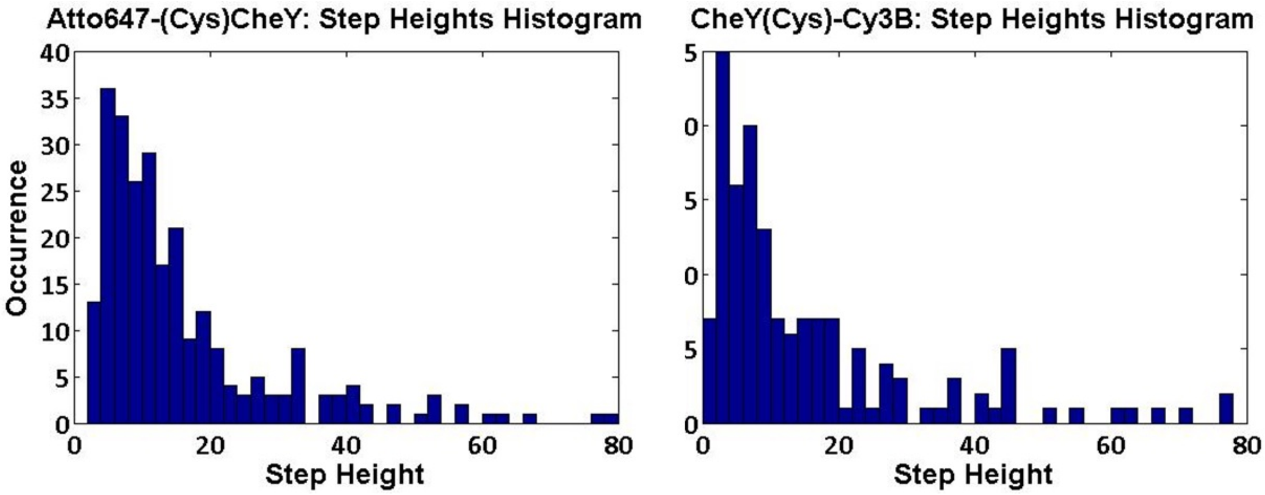

Figure S1: Photobleaching step size analysis as a measure of Cy3B and Atto647 dyes' brightness in live *E. coli*. Left: Internalisation of CheY(Cys)-Cy3B, histogram of single-step height intensities from fitted steps (45 cells). Right: Internalisation of Atto647-(Cys)CheY, histogram of single-step height intensities from fitted steps (60 cells). See main text for estimation of unitary fluorophore intensities.

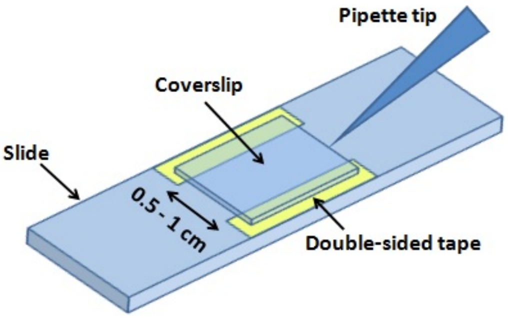

Figure S2: Schematic of a tunnel slide. The central chamber has a volume of ~25-50 µl.

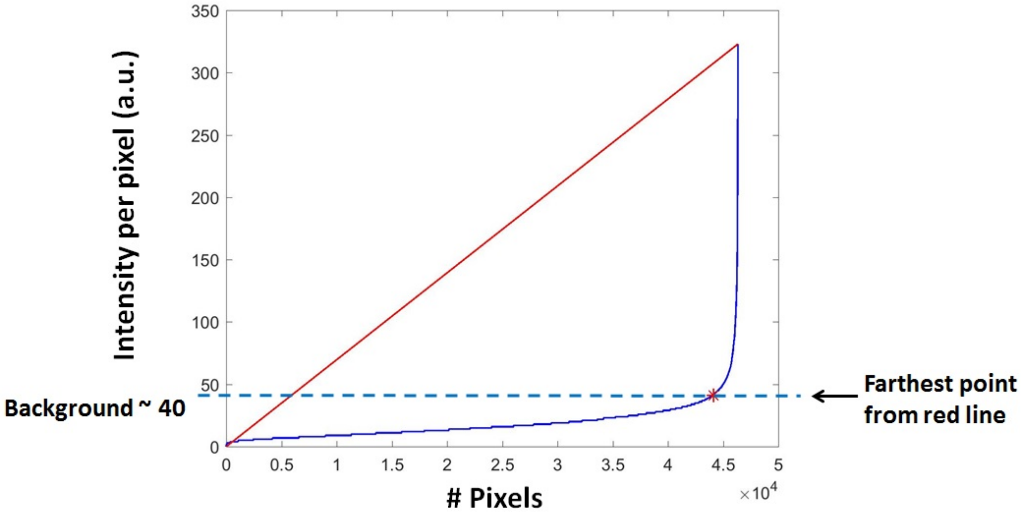

Figure S3: Fluorescence baseline subtraction in single-molecule experiments: for each frame, all pixels were sorted according to their intensities (blue curve) and the intensity value (red star) farthest away from the line (red straight line) that connects the minimum and the maximum of the sorted pixels was used for background thresholding (cyan, dashed line).

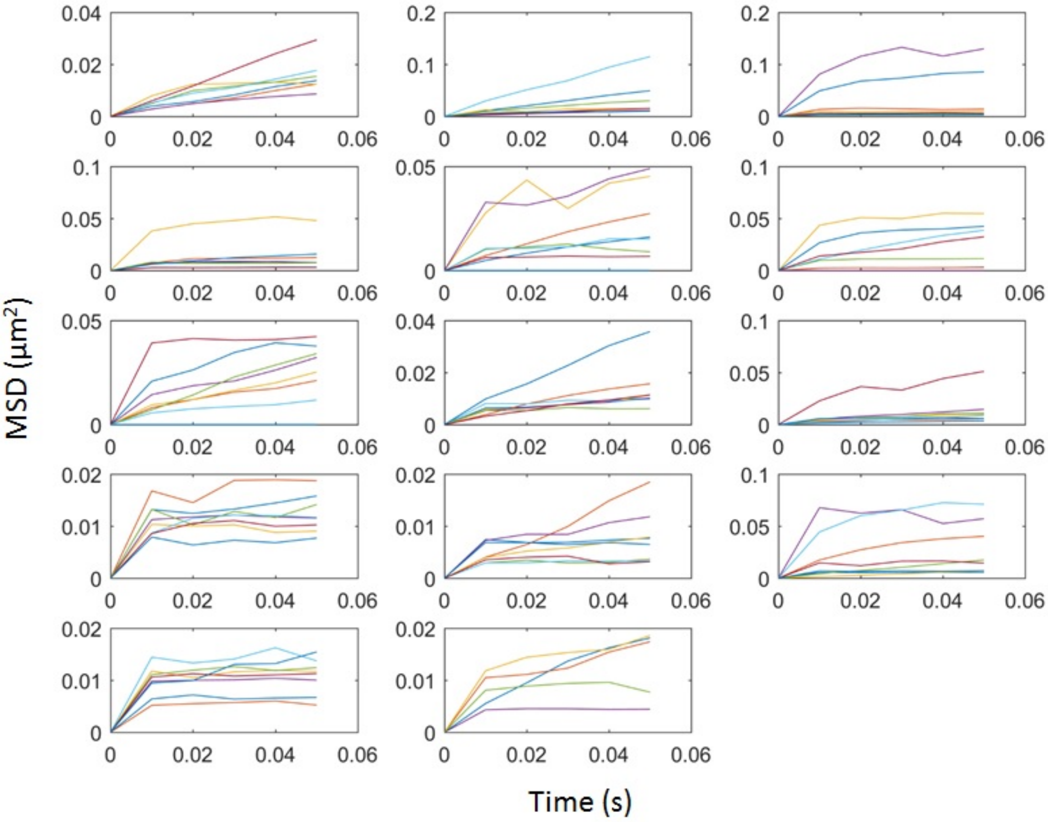

Figure S4: Mean Squared Displacement (MSD) curves over 5 frames for all tracks longer than 50 frames (108), calculated as average of the squared displacements over time lags of the same length.

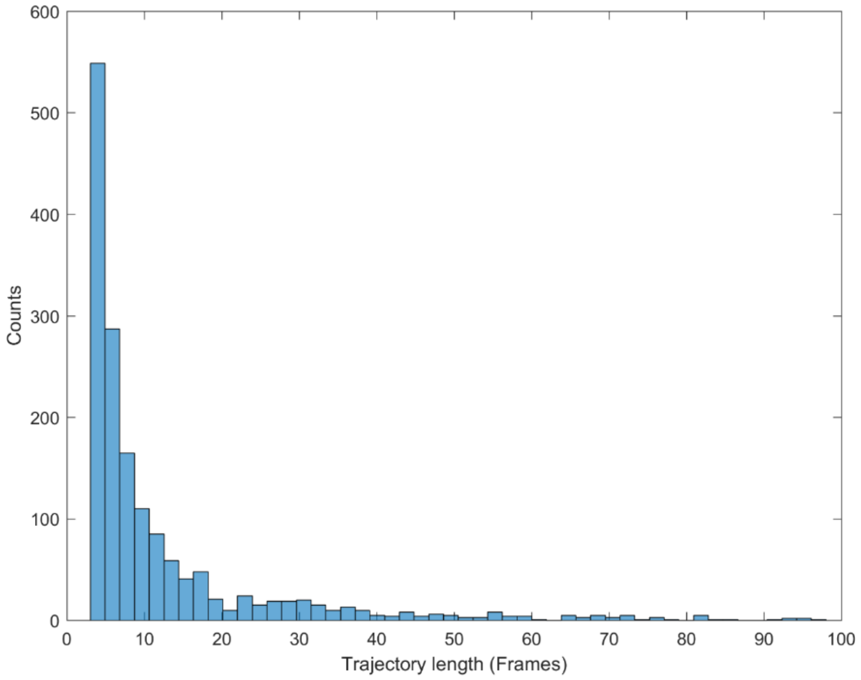

Figure S5: Distribution of lengths for all 1658 recorded trajectories. Note that due to scaling issues the tracks longer than 100 frames have been discarded from this plot; they represent about 2.6% of all the recorded tracks.

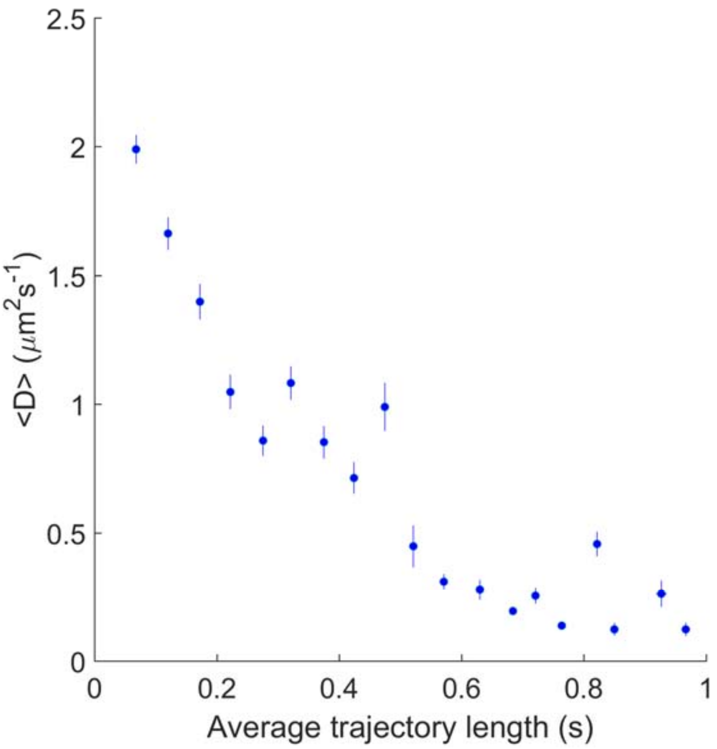

Figure S6: Average diffusion coefficient <D> versus track length. Because individual short tracks contain too few data points for a reliable estimate of D, we pooled all displacements between consecutive frames observed within tracks of a particular length, using bins of track length 5 frames wide. We calculated the mean-square displacement (MSD) in each bin, from which we derived estimates of $D = MSD/4t$, where $t = 10$ ms is the time interval between frames. Longer tracks have lower estimates of D. This may be because the requirements for long tracks in TIRF select a population of molecules that move more slowly than typical ones. Slow movement may be due to membrane association, including un-resolved binding to motors or other targets in the membrane.

Table S1: Total percentages of viable bacteria for electrocompetent E. coli RP437, FliCst cells electroporated with 0.85 µM, i.e. 17 pmoles, CheY(Cys)-Cy3B during a 3.5h time-lapse experiment on rich medium agarose pad (refer to Figure 3 in main text). As shown, the damage-minimising protocols devised in this work for both preparation of electrocompetent cells and electroporation, described in the Materials and Methods in the main text, resulted in an increase with respect to previous values reported in the literature for loaded cells [36] of both the proportion which could grow/divide after electroporation and the proportion which would remain intact but not growing up to 3.5 hours from application of the electric pulse.

**ALL Electroporated Cells (regardless of loading)**		
Growing / Dividing (%)	Not growing but intact (%)	Damaged (%)
68	20	12
**Fluorescent Electroporated Cells**		
	This work (%)	Ref. 36 in main text (%)
Growing / Dividing	38	11
Not growing but intact	43	32
Damaged	19	57

© The Authors under the terms of the Creative Commons Attribution License http://creativecommons.org/licenses/by/3.0/, which permits unrestricted use, provided the original author and source are credited.
